# The Association between Interrelationships and Linkages of Knowledge about HIV/AIDS and its Related Risky Behaviors in People Living with HIV/AIDS

**DOI:** 10.4172/2155-6113.S7-002

**Published:** 2012-09-01

**Authors:** Berhanu Tameru, Gemechu Gerbi, David Nganwa, Asseged Bogale, Vinaida Robnett, Tsegye Habtemariam

**Affiliations:** 1 Professor and Director, Tuskegee University, Center for Computational Epidemiology, Bioinformatics and Risk Analysis, 107-Williams-Bowie Hall, College of Vet Med, Nursing and Allied Health (CVMNAH), Tuskegee, AL 36832, USA; 2 Tuskegee University, Tuskegee, USA

**Keywords:** Relationships, Psychosocial factors, HIV/AIDS, Risky behaviors, People Living with HIV/AIDS (PLWHA)

## Abstract

The relationship between knowledge about HIV/AIDS and its associated risky behaviors is complex and has not been sufficiently explored. It is especially important to look at some of the aspects of this relationship among people living with HIV/AIDS (PLWHA) in order to develop effective intervention strategies to reduce HIV/AIDS risky behaviors. The objective of this study was to investigate the association between knowledge about HIV/AIDS and its risky behaviors among PLWHA.

**Methods:**

Risk taking behavior among the participants was measured as the number of all risky behaviors before and after the knowledge of their HIV/AIDS positive status. Knowledge was measured by the extent to which participants answered the HIV/AIDS related questions. The relationships between the identified HIV/AIDS risky behaviors and the knowledge among PLWHA were analyzed using Structural Equation Modelling.

**Results:**

341 questionnaires were administered and 326 (96%) were completed and returned from PLWHA clients of HIV/AIDS outreach facility in Alabama. Analysis revealed that, knowledge of HIV/AIDS, and knowledge of a properly used condom in preventing the infection through sexual activity were positively related with knowledge of where to get tested for HIV/AIDS. Using drugs before sex was significantly related with having sex with prostitutes (total effects’ standardized regression coefficient (TESRC)=0.29, p<0.001). Sharing the same syringe or needle with another person or other people to inject oneself was strongly related with number of sexual partners within one year (TESRC=0.25, p<0.001), and sex with injecting drug users (TESRC=0.45, p<0.001).

**Conclusion:**

A deeper understanding of HIV/AIDS and some of its transmission pathways appears to be very effective in practicing the taking of preventive measures such as using condoms or getting tested for HIV. Increasing access to HIV/AIDS education could therefore be very useful in providing further gains in HIV/AIDS awareness among PLWHA.

## Introduction

Emerging in the early 1980’s, Human Immunodeficiency Virus (HIV) and Acquired Immunodeficiency Syndrome (AIDS) have become serious global health challenges. Since AIDS was first diagnosed in the United States in 1981, it still remains the leading killer of humans, with over 90% of all infected cases occurring in the developing world and the number of newly infected cases rising every year [1]. The fact that lack of knowledge about HIV/AIDS leads to unprotected sexual contacts is one of the major causes of the increasing prevalence and incidence in HIV infections [1]. Although there are already many HIV/AIDS prevention efforts and expensive awareness campaigns in place to educate and disseminate the information about HIV/AIDS to the general public, these programs have not been highly successful in reducing HIV/AIDS risky behaviors. This is in the sense that, despite the vast knowledge about how HIV/AIDS is transmitted, many People Living with HIV/AIDS (PLWHA) still continue to engage in HIV/AIDS risky behaviors. This does not only put them at risk for sexually transmitted diseases including re-infection with HIV but also they place others at risk for getting infected with the HIV [2].

There is a tremendous body of evidence showing that a substantial number of PLWHA continue to engage in high-risk sexual behaviors [3]. In their study of 215 HIV-infected women living in New Jersey, Kline and VanLandingham [4] found that only 48% of the women were using condoms consistently with their primary partner. In a report from a population-based HIV risk-behavior study conducted in Los Angeles County, California, as many as 29% of a cohort of HIV infected men reported as having engaged in unprotected sexual intercourse over a previous 12 months period [5]. A study of sexual risk behaviors among heterosexual HIV discordant couples, conducted in California, found that over two-thirds of couple members surveyed reported as having have engaged in unprotected sex with their partner in the previous 6 months [6] Having learned recently about personal HIV status, multiple sex partners, low educational attainment and young age in Vietnam, were found to be associated with higher HIV risk behaviors [7].

A cross-sectional study conducted in New York found that among HIV positive women who were sexually active, 32% reported inconsistent condom use. Of those, only 37% reported that all their sexual partners were HIV-positive [8]. A study in Denmark reported a higher incidence of gonorrhea (a six-fold increase) in HIV-seropositive men who have sex with men (MSM) compared with heterosexual men, suggesting a relapse in high-risk sexual behaviors among this group [9]. In USA, a study by Ostrow and colleagues found that more than 50% of a sample of HIV-positive and HIV-negative homosexual men reported of having recently engaged in unprotected anal intercourse [9]. A large survey conducted between 1999 and 2001 in San Francisco, California, found that the proportion of MSM reporting to have had unprotected anal sex with 2 or more partners of unknown serostatus increased from 19% to 25% for HIV positive MSM, compared to an increase from 10% to 15% for HIV-negative MSM participants [10].

A study in France found that the proportion of HIV positive patients reporting risky sexual behaviors for HIV transmission, increased from 5.1% in 1998 to 21.1% in 2001–2002 [11]. In a study examining the sexual behavior of HIV discordant couples after HIV counseling and testing in Zambia, found out that 80% of the study sample reported to have used condoms. However, 32% of pregnancies and HIV transmissions were detected among couples who had reported consistent condom use [12]. Although the prevalence of HIV infection is low among MSM in Heilongjiang province, China, the situation that the risk behaviors were frequent in the population is alarming. About 48.7% of the subjects had multiple male sexual partners and only 37.3% of the subjects had consistent condom use (use every time) in the past 6 months [13].

Twenty-two percent (22%) of HIV positive MSM reported engaging in unprotected anal sexual intercourse with one or more new partners one month prior to the study [14]. A number of studies have also found that as many as one in three HIV infected people may continue to engage in unprotected sex, with sexual contacts often occurring with seronegative or of unknown HIV status partners [15,16]. The objective of this study was to assess HIV/AIDS related knowledge (awareness) and the engagement in HIV/AIDS risky behaviors and their interrelationships with each other among people living with HIV/AIDS in the Black Belt Counties of Alabama.

## Materials and Methods

### Study design

The data was collected by a survey questionnaire instrument of HIV positive clients of a Community Based HIV/AIDS Outreach Facility (CBHAOF) in Alabama. The CBHAOF provides community based HIV/AIDS treatment and prevention services through education, quality services and compassionate care to HIV/AIDS afflicted clients and their families in 27 counties in Alabama. The CBHAOF has also a medical component or clinic which provides complete primary health care that includes physician visits, and laboratory tests for diagnosing HIV infection. The questionnaire was pretested in collaboration with CBHAOF. The Tuskegee University Institutional Review Board approved the final questionnaire, informed consent forms and study protocol.

### Data collection procedures

The data was collected in collaboration with CBHAOF. A letter requesting permission of HIV positive clients to participate in the survey was sent to the executive director of CBHAOF. After the permission was granted from the executive director, the questionnaires, along with informed consent forms were given to CBHAOF staff for administration and retrieval.

The defined criteria to enroll participants in the study included: age equal to or greater than 18 years, and having been diagnosed as being HIV positive. A convenience sampling method was used to select the study sample. Eligible participants were informed about the study by the facility’s staff during their regular medical visits. Each participant was actively approached at the end of his or her visit by the trained interviewers to explain the goals of the study and request his or her consent to participate in it. Although a convenience sampling method was used, almost all the clients who were approached were eligible and agreed voluntarily to participate. Participants agreed to participate in the survey in two ways. These were by either both signing and returning the consent form before filling the questionnaire, or by directly filling the questionnaire. Participant’s names were not included in the questionnaire, thus maintaining their confidentiality and privacy.

The participants completed the questionnaire during their clinical visits to the clinic at their own convenience and that of the facility’s staff. The facility staff administered and retrieved the questionnaires at its clinical sites. A total of 341 questionnaires were distributed and 326 questionnaires were fully completed and returned (a response rate of 96%). The other 15 questionnaires (a refusal or dropout rate of 4%) were also returned but were not fully completed and as a consequence they were destroyed and were not used in the analysis.

### Measures

There are two sets of measures, the interrelationships between HIV/AIDS awareness and risky behaviors. All measures in this study were drawn from the data collected using the questionnaires.

#### The interrelationships among HIV/AIDS awareness variables

##### i) Dependent measures (variables)

The dependent variables to examine the interrelationships among HIV/AIDS awareness consisted of knowledge of where to get tested for HIV/AIDS, knowledge about Highly Active Antiretroviral Therapy (HAART), and knowledge of whether HIV is found in body fluids. For the awareness of HIV/AIDS indicators each participants was requested to answer the following questions: “Do you know where to get tested for HIV/AIDS?” “To your knowledge, are there medications available which can lengthen the life of a person infected with the HIV/AIDS?” and “Do you think HIV is found in body fluids (semen, vaginal fluids, breast milk, and blood)?” Lack of HIV/AIDS awareness was considered when a participant answered “no” to all of the questions.

##### ii) Independent measures (variables)

The independent variables included knowledge about HIV/AIDS and knowledge about whether proper condom use is in preventing HIV infection. Lack of awareness about the effectiveness of proper condom use in preventing HIV infection was assessed by asking the following question: “How effective do you think a properly used condom is in preventing infection from HIV/AIDS through sexual activity?” If the participant answered as “not at all effective”, it implied that the participant did not have enough knowledge about the effectiveness of a properly used condom is in preventing HIV infection. The responses of survey participants were analyzed to determine if there were significant relationships among HIV/AIDS education (awareness) variables.

#### Interrelationships among HIV/AIDS risky behaviors

##### i) Dependent measures (variables)

The dependent measures included sex with IDU, a participant’s number of sexual partners within one year, one month and one week before the survey was administered, needle and or syringe sharing, and sex with prostitutes. A participant with high HIV/AIDS risky behaviors was defined as that participant who answered “yes” to the following questions: “Have you ever had sexual intercourse with a person or people who inject drugs intravenously?”, “Have you ever shared the same syringe and or needle with another person to inject yourself?”, and “Have you ever had sexual intercourse with a prostitute?” Other measures of HIV/AIDS risky behaviors assessed in this study are self-reported number of sexual partners a participant had within one year, one month and one week. These were determined by asking the following questions: ‘How many people have you had sexual intercourse with in one year; one person; two people; three people; four people; five people and six or more people’. Same type and sequence of questions were asked for the one month and one week periods. A participant who responded as having had more than one sexual partner was considered to be at a higher risk.

##### ii) Independent measures (variables)

The independent measures included using drugs and alcohol before sex, and needle sharing. A participant with high HIV/AIDS risky behaviors was defined as one who answered “yes” to the following questions: “Did you use drugs before you had sexual intercourse the last time?, “Did you drink any alcoholic beverage such as beer, wine, wine coolers, or liquor before you had sexual intercourse the last time?, and “Have you ever shared the same syringe and or needle with another person to inject yourself?”. The responses of survey participants were analyzed to determine if significant interrelationships existed between HIV/AIDS education (awareness) and risk taking behaviors.

### Statistical analyses

A path analysis model [17–19] was used to examine the relationships between all the variables of the hypothesized model. The Analysis of Moment Structures (AMOS) software (version 17.0) [17,18] techniques were used to examine interrelationships among HIV/AIDs awareness and risky behaviors variables. Structural Equation Modeling (SEM) is designed to capture complex interrelationships among multiple variables by assessing the strengths of these interrelationships with respect to selected dependent variables. AMOS is designed for structural equation modeling and path analysis, as well as to perform linear regression analysis and Analysis of Variance (ANOVA). AMOS has also been used by Roh [18,19] for statistical analysis in other surveys. AMOS uses a path diagram as a model specification and displays parameter estimates graphically on a path diagram. It features an intuitive graphical interface that allows the analyst to specify models by drawing them. AMOS also has a built-in bootstrapping routine and superior handling of missing data. It is capable of accessing data from a number of sources, including MS Excel spreadsheets and Statistical Package for the Social Sciences (SPSS) databases.

## Results

The summary of the demographic and socioeconomic characteristics for the 326 participants by number and percentages in relation to their sex, race, age group, marital status, employment status, level of education and level of income are presented in table 1.

**Figure 1:**
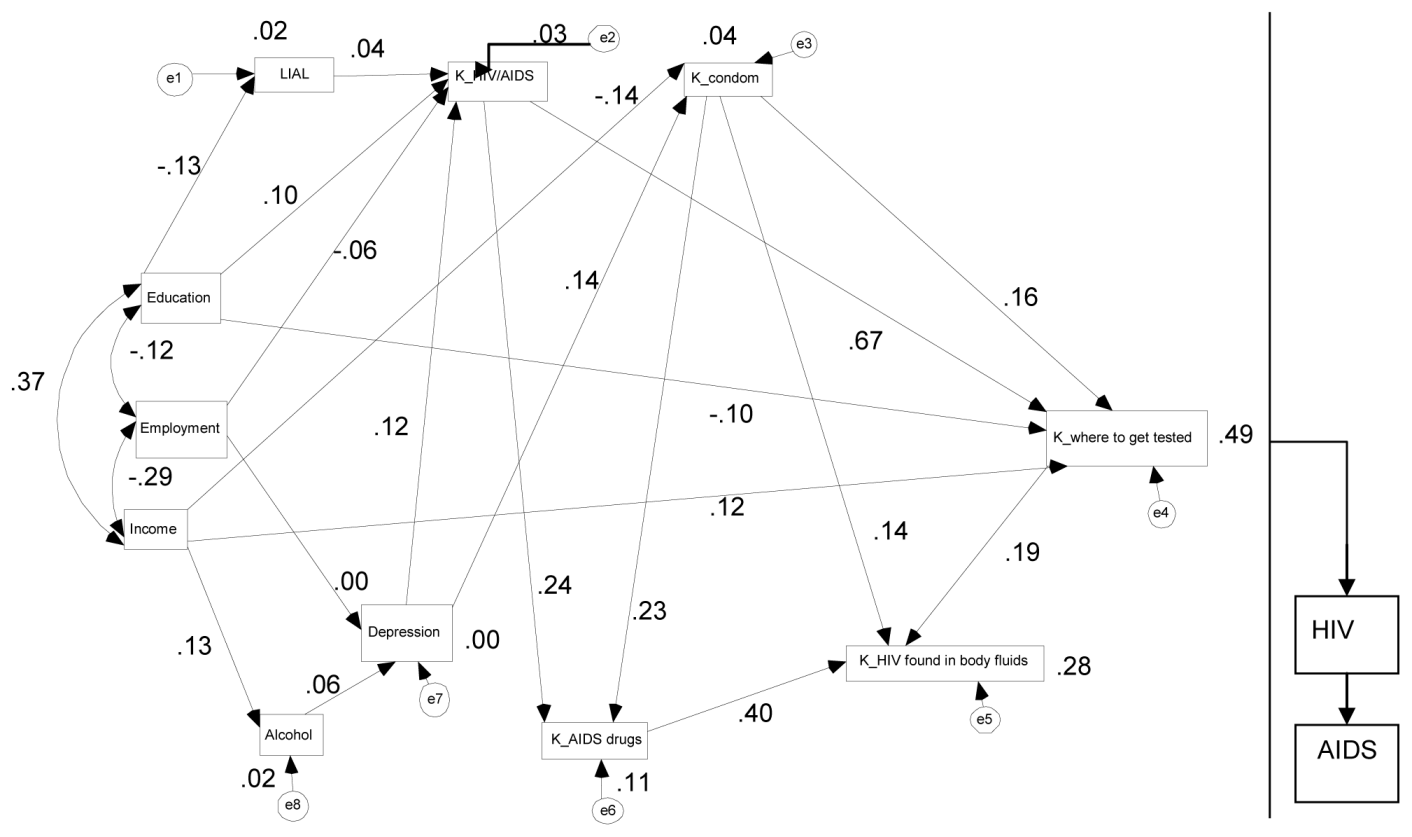
The Path Model Indicating the Direction of all Relationships Between Socioeconomic and Psychosocial Factors on HIV/AIDS Education (Awareness) Among People Living With HIV/AIDS. (Single headed arrows represent standardized regression coefficients and double headed arrows represent correlations and ei, i= 1–8, are error terms).

**Figure 2:**
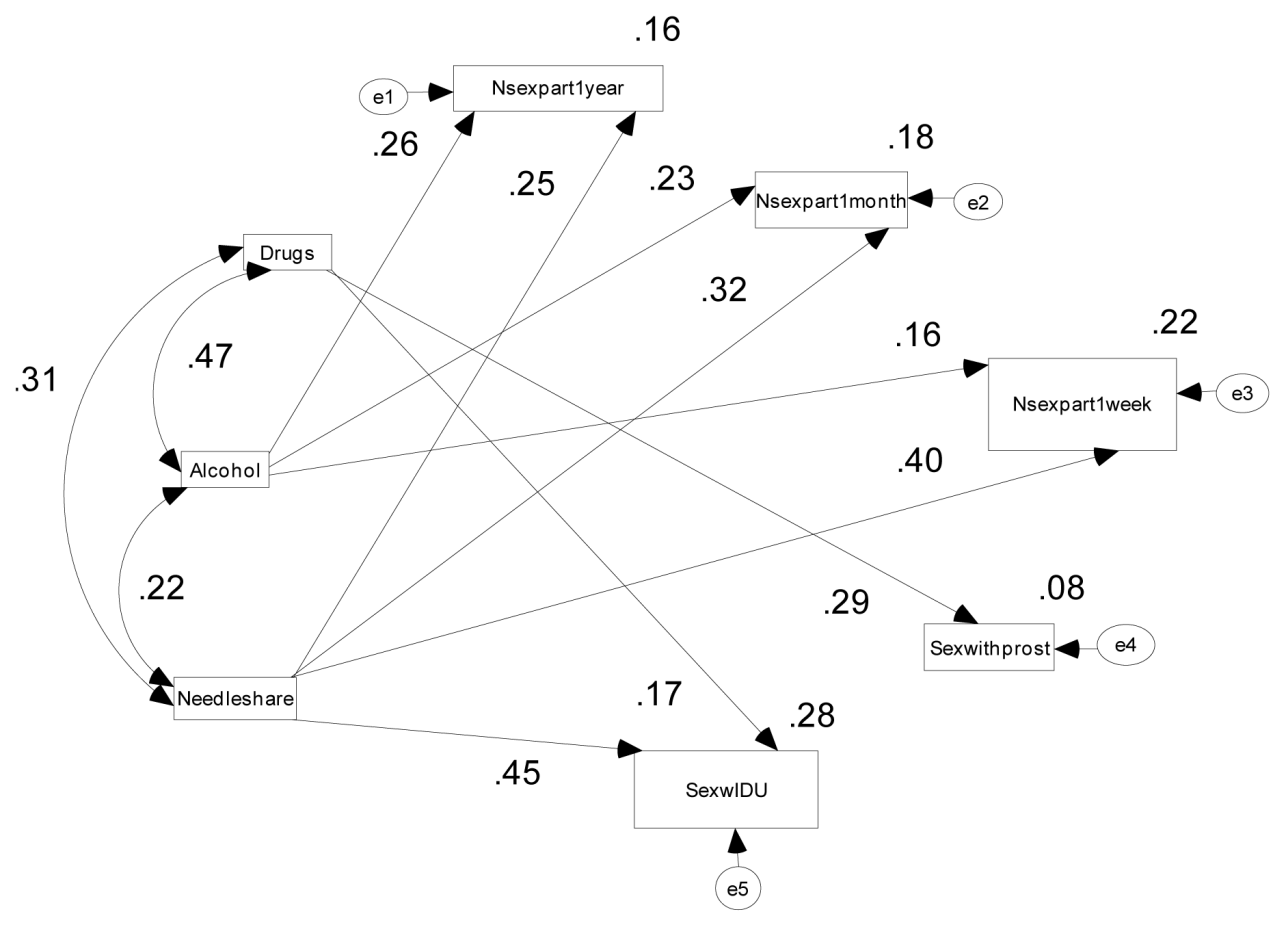
The Full Path Model indicating the direction of all interrelationships among HIV/AIDS risky behaviors. (Double headed arrows represent correlations and single headed arrows represent standardized regression paths, ei, i= 1–5, are error terms)

Structural Equation Modeling (SEM) [19,20] techniques were used to examine interrelationships among and between socioeconomic status factors, knowledge of HIV/AIDS and education (awareness) variables. SEM is able to capture complex relationships among multiple variables by assessing the strength of these interrelationships with respect to selected dependent variables. The models developed here are based on a detailed review of HIV/AIDS transmission dynamics at the human population level, with a focus on African Americans. Previous research [21] suggested that socioeconomic and psychosocial factors influence HIV/AIDS risky behaviors among African Americans. Low socioeconomic status including low levels of education, income and employment are linked to several psychosocial factors. In this study the interrelationship between HIV/AIDS risky behaviors and awareness variables were assessed. The Structural Equation Models (SEM) using AMOS version 17.0 software [17] have been developed and are presented in figures 1 and 2.

### The interrelationships among HIV/AIDS education (awareness) variables

Results of the association between HIV/AIDS knowledge variables among PLWH (Chi-Square Test for Equal Proportions) are presented in table 2. It was found out that overall, 96.9% of PLWHA have knowledge about HIV/AIDS. For the knowledge about proper condom use about 86% of PLWHA are aware of the effectiveness of proper condom usage. The other knowledge variable categories by number, percentages and P-values are presented in table 2.

Table 3 and figure 1 provide standardized regression weights for measures of HIV/AIDS education (awareness). Knowledge of HIV/AIDS transmission routes was strongly related to knowledge of where to get tested for HIV/AIDS (total effects’ standardized regression coefficient =0.67, p=<0.001) and knowledge of medications that lengthen the life of a person infected with HIV/AIDS (total effects’ standardized regression coefficient = 0.24, p = <0.001). Table 2 also shows that knowledge of a properly used condom in preventing infection from HIV through sexual activity is strongly related with: knowledge of where to get tested for HIV/AIDS (total effects’ standardized regression coefficient =0.16, p=<0.001); knowledge of medications that are available to lengthen the life of a person infected with HIV/AIDS (total effects’ standardized regression coefficient =0.23, p=<0.001); and knowledge of HIV being found in body fluids (total effects’ standardized regression coefficient =0.14, p=0.003). Table 2 further shows a statistically significant relationship between knowledge of where to get tested for HIV/AIDS and knowledge of HIV being found in body fluids (total effects’ standardized regression coefficient =0.19, p=<0.001). Knowledge of medications available to lengthen the life of a person infected with HIV/AIDS has the strongest interrelationship with knowledge of HIV being found in body fluids (total effects’ standardized regression coefficient =0.40, p=<0.001).

Figure 1 shows the standardized regression weights between SES, psychosocial variables and HIV/AIDS education (awareness) variables for significant paths (p<0.05). As shown in figure 1, three variables: knowledge of medications that are available to lengthen the life of a person infected with HIV/AIDS; knowledge of a properly used condom in preventing infection from HIV through sexual activity; and knowledge of where to get tested for HIV/AIDS were positively related with knowledge of HIV being found in body fluids. These predictors accounted for 28 percent (R^2^=0.28) of the variance in knowledge of HIV being found in body fluids (Figure 1). Levels of income, knowledge of HIV/AIDS, and knowledge of a properly used condom in preventing infection from HIV/AIDS through sexual activity were positively related with knowledge of where to get tested for HIV/AIDS. In contrast, levels of education were negatively related to knowledge of where to get tested for HIV/AIDS. These predictors accounted for 49 percent (R^2^=0.49) of the variance in knowledge of where to get tested for HIV/AIDS. When exploring the interrelationships among the predictor variables, employment status, and drinking alcohol before sex did not predict depression (Figure 1). Further analysis of the interrelationships among the predictor variables show that employment status and lost interest in aspects of life that used to be important before establishing HIV infection status did not predict knowledge of HIV/AIDS.

### Interrelationships among HIV/AIDS risky behaviors variables

Percentage distribution of the HIV/AIDS risky behavior variables is presented in table 4 and in table 5 the number of sexual partners in one month and one week is presented.

Table 6 and figure 2 indicate the standardized regression coefficients describing interrelationships among HIV/AIDS risky behaviors. As shown in table 6, using drugs before sex is significantly related with having sex with prostitutes (total effects’ standardized regression coefficient =0.29, p<0.001) and sex with IDU (total effects’ standardized regression coefficient =0.17, p<0.001). Drinking alcohol before sex is strongly related with number of sexual partners within one year (total effects’ standardized regression coefficient =0.26, p<0.001), number of sexual partners within one month (total effects’ standardized regression coefficient =0.23, p<0.001), and number of sexual partners within one week (total effects’ standardized regression coefficient =0.16, p=0.001). Table 6 and figure 2 also further indicate that sharing the same syringe and or needle with another person or other people to inject oneself is strongly related with number of sexual partners within one year (total effects’ standardized regression coefficient =0.25, p<0.001), number of sexual partners within one month (total effects’ standardized regression coefficient =0.32, p<0.001), number of sexual partners within one week (total effects’ standardized regression coefficient =0.40, p <0.001), and sex with IDU (total effects’ standardized regression coefficient =0.45, p <0.001).

## Discussion

Several components of the findings of this study that show high HIV/AIDS awareness might be of great advantage in reducing the transmission of HIV/AIDS. For example, having heard of HIV/AIDS was strongly related to knowledge of where to get tested for HIV/AIDS and the knowledge of the availability of medications to lengthen the life of a person infected with the HIV/AIDS. As indicated above, participants had high levels of HIV/AIDS awareness, but about 40% of them reported low knowledge of proper condom utilization for the prevention of HIV infection. This shows that simply educating people on HIV/AIDS may not in itself guarantee greater levels of HIV/AIDS prevention. In addition, it is necessary to examine the timing of education programs, and their design in relation to actual social conditions [22]. This suggestion follows on from the results of this study, which shows the discrepancies between a relatively high knowledge on other measures of HIV/AIDS awareness and poor knowledge of proper condom use against HIV infection. This is also consistent with what was found by [23] where misunderstanding on HIV/AIDS remains in Burundi women even having high level of awareness. In other words, knowledge about HIV/AIDS and its modes of transmission alone does not lead to prevention actions. This suggests that knowledge about HIV/AIDS may not be a sufficient condition to induce proper condom use, but it is a necessary one. The findings suggest that comprehensive knowledge of transmission, prevention and treatments of HIV/AIDS is required to adopt safer sex practices. This study has also addressed the interrelationships among HIV/AIDS awareness variables. Furthermore, participants have awareness about HIV/AIDS, modes of transmission, and treatment but they are not sufficiently aware about its prevention. This is in line with a finding in Australia where there was an extremely low level of awareness of personal risk in relation to sexually transmitted diseases and HIV [24]. This study also clearly shows that knowledge about HIV/AIDS is related to the mitigation of HIV/AIDS transmission. For example, proper condom use was significantly related to: knowledge of where to get tested for HIV/AIDS; knowledge of the availability of AIDS medications (HAART) which can lengthen the life of a person infected with the HIV; and knowledge of HIV being found in body fluids. The data from the HIV/AIDS awareness assessment suggest significant relationship between knowledge of where to get tested for HIV/AIDS and knowledge about HIV being found in body fluids. Furthermore, deeper understanding of HIV/AIDS and its transmission appears very effective in formulating and taking preventive measures such as using condoms properly or getting tested for HIV. Increasing access to HIV/AIDS education could therefore be very useful in providing further gains in HIV/AIDS awareness among PLWHA. Further studies are required to determine the extent to which dissemination of HIV/AIDS knowledge using specific media automatically translates into conversations with the sexual partner about personal risk.

In terms of demography, age was found to be significantly negatively related with the knowledge of the effectiveness of properly used condoms in preventing infection with the HIV through sexual activity, knowledge of medications available to lengthen the life of a person infected with HIV/AIDS, and knowledge that HIV is found in body fluids. Gender is significantly related with knowledge of where to get tested for HIV/AIDS with males having a higher rate compared to females (58% for males versus 42% for females). Race is significantly related to having have heard of HIV/AIDS, knowledge of where to get tested for HIV/AIDS; the knowledge of medications available to lengthen the life of a person infected with the HIV/AIDS; and knowledge that HIV is found in body fluids. Whites (non-Hispanic) were more likely to report having heard of HIV/AIDS compared with African Americans. In contrast, African Americans were more likely to have the knowledge of where to get tested for HIV/AIDS and knowledge that HIV is found in body fluids. Other races such as Native Americans accounted for the significant relationships observed between races with respect to the knowledge of medications available to lengthen the life of a person infected with HIV/AIDS.

Participants who were engaged in at least one HIV/AIDS risky behavior were more likely to be engaged in other risky behaviors. Specifically substance use (drugs and alcohol) is significantly related with high HIV/AIDS risky behaviors including sex with prostitutes, sex with injecting drug users and multiple sexual partners. In the literature, there is a general consensus that alcohol and/or drug use heighten the probability of sexual impulsivity contributing to HIV infection. Consistent with these findings, there are also drug and alcohol studies that have examined specific variables related to sexual risk taking, predominantly in non-HIV infected people. For example, a study noted that drug users were more likely to report as having had sex in exchange for money and/or drugs, sex with an injection-drug user, and a history of having contracted a sexually transmitted disease [25].

Studies have also confirmed relationships between substance use and high-risk sexual behaviors [22,26] including multiple partners, and transactional sex (i.e., sex for money) in China [27]. These types of relationships have been examined in other studies as well. For example, Breen and colleagues [28] found that alcohol use was related to sex with a greater number of partners. In addition, Stein and colleagues [29] found out that among intravenous drug users with alcohol problems, alcohol use predicted risk-taking behaviors with regard to sex. Enhanced sexual impulsivity has been reported with drugs other than alcohol as well. For example, Raj and colleagues [30] found among detoxification patients that cocaine use was associated with being sexually active as well as transactional sex.

Clearly, the data confirm relationships between alcohol and/or drug use and specific sexual related risky behaviors. The findings of this study suggest that PLWHA are faced with many pressures and decisions regarding drugs, alcohol, and sexual activity and that these decisions are more likely to occur simultaneously. When considering the different aspects of HIV/AIDS risky behaviors, it is important to realize the impact of drugs and alcohol on decision-making. Future studies are needed to clarify what happens to peoples thought processes when they use drugs and drink alcohol and to explore the relationships between alcohol/drug usage and sexual impulsivity, in addition to examine the role of personality as a moderating variable. As stated earlier, since alcohol affects judgment and lowers inhibitions, people sometimes do things when they use drugs and drink alcohol that they would not normally do. This can include having multiple sexual partners, sex with prostitutes and injecting drug users when they normally would not practice these risky behaviors. These findings suggest that PLWHA should be educated to practice safe behaviors by not letting drugs and alcohol impair their decision-making process in their encounters with their sexual partners.

Much of the research on HIV has focused on individual-level risk factors. Although these factors play a role in the transmission of the virus, they do not fully explain the variance in HIV transmission among different racial and ethnic groups. Since individual behaviors take place in social contexts, future research and interventions related to HIV must focus more keenly on ecological variables, such as the community-level, relationship-level, and biological-level variables. Specifically, it is useful to address some types of individual-level risk factors including knowledge, motivation, and behavioral intentions.

## Conclusion

In the current study it was observed that better knowledge of HIV/AIDS and its transmission mechanisms have not been shown to have a positive relationship to the knowledge of HIV/AIDS prevention. Levels of education is negatively related with lost interest in aspects of life that used to be important before establishing HIV infection status and levels of income is positively related with drinking alcohol before sex. These findings suggest that psychosocial problems among PLWHA are best understood within a combined effect of levels of education and income.

Incorporating the psychosocial variables in assessing sexual risk behavior in people living with HIV/AIDS could be helpful in formulating public policies and prevention strategies for HIV/AIDS prevention. Since HIV/AIDS is spread most often through unprotected sex with an infected partner, this study presents some of the behavioral, social, and psychological factors that influence unprotected sex. Factors that predicted unprotected sex are lack of basic knowledge about HIV/AIDS transmission and prevention, lack of awareness of their partner’s HIV status, problems communicating about safer sex, attributing their HIV infection to something that another person intentionally did it to them, anxiety, depression and less intention/commitment to use condoms and safer sex. The findings illustrate the types of strategies that need to be addressed in behavioral interventions for PLWHA.

## Limitations

Limitations include the use of a clinic-based convenience sample study of 326 PLWHA in Alabama, USA, which means that the findings presented here may not be generalizable to the broader population of PLWHA. Secondly, the study relied on self-report measures of sensitive issues like knowledge about condom use, number of sexual partners and drug/substance use before sex. Thus, it is likely that high or lower awareness or high-risky behaviors leading to HIV infection are underreported or over reported in the data we used.

## Figures and Tables

**Table 1:** Demographic and socioeconomic characteristics of the participants by number and percentages.

Demographic and social economic characteristics		n	%
Sex	Female	136	42
	Male	181	56
	Transgender	4	1
	Transsexual	5	2
Race	African American	208	64
	White (non-Hispanic)	94	29
	Hispanic	10	3
	Other races	14	4
Age group	18–29	53	19
	30–39	86	30
	40–49	104	37
	50–59	34	12
	60 and above	6	2
Marital Status	Single	183	56
	Married	47	15
	Divorced	47	15
	Separated	31	10
	Widow(er)	3	1
	Other	13	4
Employment Status	Employed for wages	122	39
	Unable to work	59	19
	Unemployed	50	16
	Student	25	8
	Homemaker	25	8
	Self-employed	18	6
	Retired	12	4
Level of education	Graduate school	11	3
	College 4 years or more	50	15
	College 1 year to 3 years	85	26
Level of income	$9,999 or under	97	31
	$10,000 to $14,999	45	14
	$15,000 to $19,999	38	12
	$20,000 to $24,999	36	11
	$25,000 to $29,999	23	7
	$30,000 to $49,999	20	6
	$50,000 to $74,999	13	4
	Don’t know	46	14

**Table 2:** HIV/AIDS Knowledge Variables by number and percentage and Chi-Square Test for Equal Proportions.

Variable	n	%	P-value
**Knowledge about HIV/AIDS (***K_HIV/AIDS)*			<.0001
Yes	313	96.9	
No	10	3.1	
**Knowledge about condom***(K_condom)*			<.0001
Very effective	191	59.5	
Somewhat effective	85	26.48	
Not at all effective	17	5.3	
Don’t know	28	8.72	
**Knowledge of where to get tested**(*K_where to get tested*)			<.0001
Yes	307	95.94	
No	13	4.06	
**Knowledge about AIDS drugs**(*K_AIDS drugs*)			<.0001
Yes	267	83.44	
No	11	3.44	
Don’t know	42	13.13	
**Knowledge about HIV found in body fluids** (*K_HIVfound in blood fluids* )			**<.0001**
Yes	265	82.55	
No	10	3.12	
Don’t know	46	14.33	

Due to missing responses, some variable categories do not add up to 326. P-values are based on Pearson’s Chi-Square test between each of the knowledge variables.

**Table 3:** The interelationships among HIV/AIDS Knowledge Variables by Total Regression Effects and p-Values.

	Total effects	p-Value
The relationship between: Knowledge of HIV/AIDS transmission routes (*K_HIV/AIDS*) and knowledge of where to get tested for HIV/AIDS (*K where to get tested*)	0.67	<0.001
Knowledge of HIV/AIDS and knowledge of AIDS drugs (*K_AIDS drugs*)	0.24	<0.001
Knowledge about condom use (*K_condom*) and knowledge of AIDS drug	0.23	<0.001
Knowledge about condom use (*K_condom*) and knowledge about HIV as being found in body fluids	0.14	0.003
Knowledge of AIDS drugs (*K_AIDS drugs*) and knowledge about HIV being found in body fluids (*K_HIVfound in blood fluids* )	0.40	<0.001

**Table 4:** HIV/AIDS Risky behavior variables (N = 326) by number and percentage.

Variable	n	%
**Drinking alcohol before sex**		
Yes	90	36.59
No	156	63.41
**Drugs before sex**		
Yes	43	17.55
No	181	73.88
Don’t Know	21	8.57
**Sex with a prostitute**		
Yes	43	17.13
No	167	66.53
Don’t Know	41	16.33
**Sex with IDU**		
Yes	39	15.54
No	140	55.78
Don’t Know	72	28.69

Due to missing responses, some variable categories do not add up to 326.

**Table 5:** Number of sexual partners within a month and a week by number and percentages.

Number of sexual partners a participant had sex with in	0	1	2	3	4	5	6	7
**one month**								
*n*	32	130	35	15	10	6	18	80
*%*	9.8	39.9	10.7	4.6	3.1	1.8	5.5	24.5
**one week**								
*n*	35	124	26	12	5	6	12	106
*%*	10.7	38.0	8.0	3.7	1.5	1.8	3.7	32.5

**Table 6:** The interrelationships of HIV/AIDS risky behavior variables among PLWHA

Variable	Total effects Standardized Regression Coefficients (SRCs)	p-Value
The relationship between using drugs before sex and having sex with prostitutes (*Sexwithprost*)	0.29	<0.001
The relationship between using drugs before sex and sex with IDU (*SexwIDU*)	0.17	<0.001
The relationship between drinking alcohol before sex (*Alchol*) and number of sexual partners within 1 year (*Ndrxpart1year*)	0.26	<0.001
The relationship between drinking alcohol before sex (*Alchol*) and number of sexual partners within 1 month (*Ndrxpart1month*)	0.23	<0.001
The relationship between drinking alcohol before sex and number of sexual partners within 1 week	0.16	0.001
The relationship between needle sharing (*Needleshare*) and number of sexual partners within 1 year (*Ndrxpart1year*)	0.25	<0.001
The relationship between needle sharing and number of sexual partners within 1 month	0.32	<0.001
The relationship between needle sharing and number of sexual partners within 1 week	0.40	<0.001
The relationship between needle sharing and sex with IDU	0.45	<0.001

## References

[1] Luber AD, Kodakimble MA, Young LL, Kradjan WA, Guglielmo J (2002). Pharmacotherapy of HIV infection. Applied Therapeutics.

[2] (2001). AIDS epidemic update–December.

[3] Schiltz MA, Sandfort TG (2000). HIV-positive people, risk and sexual behaviour. Soc Sci Med.

[4] Kline A, VanLandingham M (1994). HIV-infected women and sexual risk reduction: the relevance of existing models of behavior change. AIDS Educ and Prev.

[5] Eich-Höchli D, Niklowitz MW, Clement U, Lüthy R, Opravil M (1998). Predictors of unprotected sexual contacts in HIVinfected persons in Switzerland. Arch Sex Behav.

[6] van der Straten A, Gomez CA, Saul J, Quan J, Padian N (2000). Sexual risk behaviors among heterosexual HIV serodiscordant couples in the era of post-exposure prevention and viral suppressive therapy. AIDS.

[7] Thanh DC, Hien NT, Tuan NA, Thang BD, Long NT (2009). HIV risk behaviours and determinants among people living with HIV/AIDS in Vietnam. AIDS Behav.

[8] Wilson TE, Minkoff H (2001). Brief report: Condom use consistency associated with beliefs regarding HIV disease transmission among women receiving HIV antiretroviral therapy. J Acquir Immune Defic Syndr.

[9] Ostrow DE, Fox KJ, Chmiel JS, Silvestre A, Visscher BR (2002). Attitudes towards highly active antiretroviral therapy are associated with sexual risk taking among HIV-infected and uninfected homosexual men. AIDS.

[10] Chen SY, Gibson S, Katz MH, Klausner JD, Dilley JW (2002). Continuing increases in sexual risk behavior and sexually transmitted diseases among men who have sex with men: San Francisco, Calif, 1999–2001, USA. Am J Public Health.

[11] Desquilbet L, Deveau C, Goujard C, Hubert JB, Derouineau J (2002). Increase in at-risk sexual behaviour among HIV-1- infected patients followed in the French PRIMO cohort. AIDS.

[12] Allen S, Meinzen-Derr, Kautzman M, Zulu I, Trask S (2003). Sexual behavior of HIV discordant couples after HIV counseling and testing. AIDS.

[13] Liu S, Wang K, Yao S, Guo X, Liu Y (2010). Knowledge and risk behaviors related to HIV/AIDS, and their association with information resource among men who have sex with men in Heilongjiang province, China. BMC Public Health.

[14] Stephenson JM, Imrie J, Davis M, Mercer D, Black C (2003). Is use of antiretroviral therapy among homosexual men associated with increased risk of transmission of HIV infection?. Sexually Transm Infect.

[15] Kalichman SC, Weinhardt LS, DiFonzo K, Austin J, Luke W (2002). Sensation seeking and alcohol use as markers of sexual transmission risk behavior in HIV-positive men. Ann Behav Med.

[16] Semple SJ, Patterson TL, Grant I (2000). Partner type and sexual risk behavior among HIV positive gay and bisexual men: social cognitive correlates. AIDS Educ Prev.

[17] Arbuckle James L, Werner W (1999). AMOS user’s guide.

[18] Roh Tae Hyup, Ahn Cheol Kyung, Han Ingoo (2005). The priority factor model for customer relationship management system success. Expert Systems with Applications.

[19] Kline Rex B (2010). Principles and practice of structural equation modeling.

[20] Mueller RO (1996). Basic Principles of Structural Equation Modeling.

[21] O’Leary A, Jemmott LS (1995). Women at Risk: Issues in the Primary prevention of AIDS. General issues in the prevention of AIDS in women.

[22] Agha S (2003). The impact of a mass media campaign on personal risk perception, perceived self-efficacy and on other behavioural predictors. AIDS Care.

[23] Ngayimbesha A, Chen PJ (2011). AIDS awareness among women and its influence on attitude toward people living with HIV/AIDS in Burundi. East Afr J Public Health.

[24] Fagan P, McDonell P (2010). Knowledge, attitudes and behaviours in relation to safe sex, sexually transmitted infections (STI) and HIV/AIDS among remote living north Queensland youth. Aust N Z J Public Health.

[25] Molitor F, Truax SR, Ruiz JD, Sun RK (1998). Association of methamphetamine use during sex with risky sexual behaviors and HIV infection among non-injection drug users. West J Med.

[26] Fierros-Gonzalez R, Brown JM (2002). High risk behaviors in a sample of Mexican American college students. Psychol Rep.

[27] Lin D, Li X, Yang H, Fang X, Stanton B (2005). Alcohol intoxication and sexual risk behaviors among rural-to-urban migrants in China. Drug Alcohol Depend.

[28] Breen C, Degenhardt L, Kinner S, Bruno R, Jenkinson R (2006). Alcohol use and risk taking among regular ecstasy users. Subst Use Misuse.

[29] Stein D, Anderson B, Charuvastra A (2001). Alcohol use and sexual risk taking among hazardously drinking drug injectors who attend needle exchange. Alcohol Clin Exp Res.

[30] Raj A, Saitz R, Cheng M, Winter M, Samet JH (2007). Associations between alcohol, heroin, and cocaine use and high risk sexual behaviors among detoxification patients. Am J Drug Alcohol Abuse.

